# Does anthracycline-based chemotherapy in pregnant women with cancer offer safe cardiac and neurodevelopmental outcomes for the developing fetus?

**DOI:** 10.1186/s12885-017-3772-9

**Published:** 2017-11-21

**Authors:** Marialuisa Framarino-dei-Malatesta, Paolo Sammartino, Angela Napoli

**Affiliations:** 1 Department of Gynecologic Obstetrics and Urologic Sciences, University Sapienza Rome, Viale del Policlinico, 155 00161 Rome, Italy; 2 Department of Surgery “Pietro Valdoni”, University Sapienza Rome, Viale del Policlinico, 155 00161 Rome, Italy; 3 Department of Clinical and Molecular Medicine, University Sapienza Rome, Via di Grottarossa 1035/1039, 00189 Rome, Italy; 4 Italian Diabetic and Pregnancy Study Group, Rome, Italy

**Keywords:** Anthracyclines, Cancer pregnancy, Anthracycline cardiotoxicity, Neurodevelopmental outcomes

## Abstract

**Background:**

Cancer treatment during pregnancy is a growing problem especially now that women delay childbearing. Systemic treatment of these malignancies during pregnancy centers mainly on the anticancer drugs anthracyclines, widely used in treating hematological and breast cancer during pregnancy and sometimes associated with early and late toxicity for the fetus. Owing to concern about their cardiac and neurodevelopmental toxicity more information is needed on which anthracycline to prefer and whether they can safely guarantee a cardiotoxicity-free outcome in the fetus.

**Discussion:**

The major research findings underline anthracycline-induced dose-dependent effects, including cardiotoxicity, many avoidable. Partly because the placenta acts mainly as a barrier, research findings indicate low transplacental anthracycline transfer. Anthracycline-induced teratogenicity depends closely on when patients receive chemotherapy. Anthracycline cardiac toxicity may depend on the association with drugs that inhibit or induce placental P-glycoprotein (P-gp). P-gp-induced drug interactions may alter placental P-gp barrier function and subsequently change fetal exposure. Though many anthracyclines have acceptable safety profiles clinical studies suggest giving idarubicin with special caution. Patients and doctors who care for pregnant women should whenever possible avoid prematurity and hence reduce prematurity-induced medical complications at birth and in the long-term. Information is lacking on long-term anthracycline-induced effects.

**Conclusion:**

Pregnant women receiving anthracycline-based chemotherapy should undergo regular, state-of-the-art diagnostic imaging to detect fetal drug-induced cardiac damage early, and allow alternative therapeutic options. Recognizing drug-induced interactions and understanding the most vulnerable fetuses will help in choosing tailored therapy. Future research on placental transport, blood-brain barrier drug passage and pharmacokinetics will improve the way we manage these difficult-to-treat patients and their fetuses.

## Background

Though cancer during pregnancy is a rare event, affecting only 1 in 1000 to 1 in 1500 pregnancies [1], deciding on anticancer drug treatment options for pregnant women is a challenging task. Given the current general trend for women in developed countries to delay childbearing, treating cancer in pregnant women is also a growing problem [2].

Breast cancer in pregnancy accounts for 0.7% of all breast cancer cases, as reported in studies from western countries [3, 4]. Hematological cancer**s** are less frequent than breast cancer in pregnancy [5].

Systemic treatment of these malignancies during pregnancy centers mainly on the anticancer drugs anthracyclines. Anthracyclines are widely used in treating hematological and breast cancer during pregnancy and sometimes associated with early and late toxicity for the fetus.

Up-to-date, comprehensive information is needed on which anthracycline to choose for treating pregnant women with cancer, how to use these potentially teratogenic medications during pregnancy, and how to reduce the risks of fetal toxicity. In this overview, to help gynecologists, oncologists and obstetricians who take difficult decisions on anticancer therapy for pregnant patients we assess major research findings on placental drug transfer, consider possible teratogenicity and cardiotoxicity and suggest directions for future research.

## Methods

In this review, we searched PubMed and Medline for English language papers focusing on anthracycline use for cancer in pregnancy published from 1975 to 2011 using the search terms anthracyclines, transplacental anthracycline transfer, cancer in pregnancy, anthracycline cardiac toxicity, and anthracycline-related neurodevelopmental toxicity. We included research articles, reviews, case reports and consensus statements. Potential, relevant studies were further identified by the article title and abstract and then assessed in detail.

## Discussion

### Through what anticancer mechanisms do anthracyclines act?

Anthracyclines act against widely ranging solid tumors and hematological malignancies by inducing complex and multiple patterns of DNA damage in anthracycline-treated cancer cells [6]. Anthracycline-induced cytotoxic activity involves multiple pathways: one is topoisomerase II inhibition, induced when the 9,10- anthraquinone ring intercalates between adjacent DNA base pairs causing the formation of single and double-strand protein-associated DNA [7]. By interacting with the topoisomerase-II-beta enzyme, impaired DNA repair has an important role in modulating DNA double helix supercoiling, and produces hydroxyl radicals (OH-**)** causing antitumor effects and toxicity to healthy tissues [8].

Some evidence suggests that these radicals are produced via poly (ADP-ribose) polymerase (PARP) and nicotinamide adenine dinucleotide phosphate (NADPH) oxidase causing the caspase hyperactivation that is essential for cell apoptosis or programmed cell death [9]. Despite their anticancer activity, anthracycline tolerability is limited by cardiotoxicity, a dose-dependent effect that can cause irreversible cardiomyopathy and congestive heart failure.

Given that cardiomyocytes do not replicate, anthracycline presumably induces dose-dependent cardiotoxicity, which can cause irreversible heart failure through molecular mechanisms other than DNA synthesis. Because reducing mitochondrial iron levels protects against doxorubicin (DOX)-induced cardiomyopathy its cardiotoxicity could reflect interactions with cellular iron metabolism causing mitochondrial iron accumulation [10]. Differences in the ability of some iron chelators in lowering DOX-induced cardiotoxicity could reflect their varying effectiveness in decreasing mitochondrial iron [11].

**Fig. 1:**
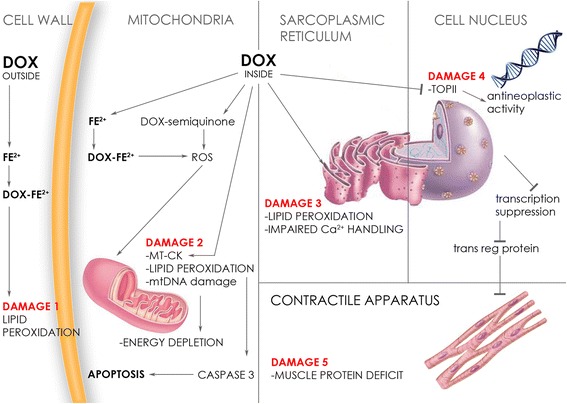
The mechanism underlying anthracycline-induced myocyte toxicity. DOX: doxorubicin; MT-CK: mitochondrial creatine kinase; Fe^2+^: iron; ROS: reactive oxygen species; Ca^2+^: calcium; TOPII: topoisomerase II; trans reg protein: transcriptional regulatory protein. Outside the cell DOX forms iron anthracycline complexes inducing membrane lipid peroxidation. After entering into the cell, DOX by interacting with the topoisomerase-II-beta enzyme, impairs DNA transcription, sarcoplasmic reticulum and muscle proteins. ROS production interacts with cellular iron metabolism causing mitochondrial iron accumulation and toxicity to mitochondrial functioning

The mechanisms responsible for anthracycline-induced cardiotoxicity also include altered cell membrane fluidity and ion transport generating reactive oxygen species (ROS) from iron anthracycline complexes, leading to lipid peroxidation and membrane damage [12]. Fibrillar and structural protein network remodeling involving the so-called extracellular matrix activated by anthracycline-induced activity contributes to cardiac damage [13] (Figure 1).

Two specific developmental features explain why anthracycline induces cardiotoxicity in the fetus. First, fetal cardiac tissue is especially vulnerable to free radical damage owing to low cardiomyocyte tolerance to changes in the oxidant-antioxidant system [14]. Second, the fetal myocardium is especially vulnerable to chemotherapy-induced damage because embryonic and fetal myocytes are smaller than those in adults and their force-generating ability increases only over time during prenatal life when contractile proteins develop and undergo structural organization [15].

### Placenta: Barrier or filter? Hints from pharmacokinetics

The extent of placental transfer and fetal drug concentration depend on the molecular weight, physicochemical drug properties such as lipid solubility, maternal concentration, fetal drug clearance, differences in drug binding protein and the pH at which the drug is ionized. Low-molecular-weight (< 500 Da), lipid-soluble and unbound compounds can easily cross the human placenta [16].

Even though the physical and chemical drug characteristics can drive transplacental passage, observations in recent years attribute placental permeability to various active efflux transporters such as P-glycoprotein (P-gp), a 170 kDa membrane protein belonging to the ATP-binding cassette superfamily, highly expressed in apical syncytiotrophoblast [17].

Through the energy supplied by ATP hydrolysis, P-gp acts as a pump able to remove drugs from the cell membranes and cytoplasm thus preventing cytotoxic compounds present in the maternal circulation from entering the placenta [18]. The breast cancer resistance protein (BCRP), another active transporter, expressed at the apical surface of the syncytiotrophoblast, also transports many substrates including anthracyclines and other chemotherapeutic agents [19]. In the large group of ATP-binding cassette transporters with 52 members, 3 other proteins are heavily involved in drug transport: ABCC1,2, 3 or MRP 1,2,3 [20, 21]. Their expression profile varies with advancing gestation [22], transcription factors, steroid hormones [23], and inflammatory pathways [24]. Genetic variations in the genes encoding these drug transporters modulate drug transporter expression [25]. Drug transporter protein expression and activity in the placenta may change during gestation and is tightly regulated by many factors. In syncytiotrophoblasts, transporters are found both in the apical membrane facing maternal blood and basolateral membrane close to fetal capillaries. The human placenta is nevertheless structurally complex and in vitro models may not overlap with human models owing to the morphologically heterogenous placentas in the various species and in their differing membrane transporter expression. Hence placental drug transporters need more detailed preclinical and clinical studies. Current evidence therefore implies that the placenta acts mainly as a barrier and less as a filter***.***

### Transplacental anthracycline transfer

Studies designed to assess fetal exposure to chemotherapeutic drugs including anthracyclines have reported their transplacental transfer from mother to fetus. In vivo data on transplacental drug transfer in pregnant women only partly overlap in vitro published data owing to interindividual genetic and metabolic differences responsible for pharmacokinetic and pharmacodynamic variabilities in pregnancy.

The first studies on transplacental passage of DOX and epirubicin (EPI) date back to 1975 when these drugs were introduced for treating various neoplastic disorders, in particular breast and hematological cancers [26]. Because all the anthracyclines except idarubicin have a molecular weight larger than 500 Da and high plasma protein binding (50–85%), they presumably have a low transplacental passage. DOX given at low doses resulted in undetectable drug levels in amniotic fluid, fetal brain or gastrointestinal tract [27] or in cord blood from a healthy child born 48 h after treatment [28]. Neither DOX nor its metabolites were detected in amniotic fluid, collected through amniocentesis in a 31-year-old woman at 28 weeks treated weekly with DOX chemotherapy [29]. DOX achieved low global transfer value**s** (2.96% **±** 0.75%) and seemed not to be dose-dependent also in vitro studies [30]. The mean EPI transfer value through the human placenta perfused at fetal and intervillous spaces by Earle’s liquid was even lower than that for DOX showing less placental toxicity than DOX [31]. Daunorubicin was found at low concentration**s** in liver, kidney and lung from a dead fetus from a mother with acute myeloid leukemia [32].

Kinetic studies designed to reproduce the placental circulation in women began on animal models. Transplacental transfer of DOX, EPI, and daunomycin was reproduced in a mouse model showing that fetal anthracycline levels were much lower than maternal levels (5.1 ± 0.6%, 4.8 ± 3.8%, 13.3 ± 3.5%) [33]. Fetal blood samples from pregnant rats receiving DOX contained a plasma concentration 6.2% that of the mother. Hence fetal DOX exposure left heart microstructure or cellular DNA turnover and apoptosis unchanged [34].

Subsequently, transplacental passage of anthracyclines in animal models was studied for the most frequently applied combination regimens. The milestone in this research remains the collection of fetal and maternal blood samples, amniotic fluid, urine, fetal and maternal tissues, and cerebrospinal fluid in the baboon pregnant model treated with fluorouracil–EPI–cyclophosphamide (FEC) and DOX–bleomycin–vinblastine–dacarbazine (ABVD). Fetal plasma concentrations of DOX and EPI averaged 7.5 ± 3.2% and 4.0 ± 1.6% of maternal concentrations [35].

Unlike other anthracyclines, idarubicin is more lipophilic and a less avid substrate for P-gp, chemical properties that may explain its higher efficacy in chemoresistant tumors [36] and greater transplacental transfer. This biochemical feature probably involves a lack of a host defense mechanism against this harmful substance causing fetal toxicity.

The clinically approved liposomal DOX formulations, non-pegylated (PL) liposomal DOX and PL liposomal DOX, were introduced to reduce anthracycline-induced adverse effects [37]. Human placental perfusion studies showed that like DOX*,* non-PL liposomal DOX has low transfer kinetics from maternal to fetal circulation whereas PL-DOX proved undetectable in the fetal circulation being extremely low in the perfused placental tissue [38].

These research results indicating low transplacental anthracycline transfer prompted clinical studies to corroborate previous findings.

### Anthracyclines in human pregnancy

#### Teratogenic risks

In general, the teratogenic effect of chemotherapeutics depends on several factors including the stage of embryonic development. From conception until implantation (2 weeks) insults to the embryo are likely to result in death and miscarriage or in intact survival because the embryo is undifferentiated and damage can be repaired as remaining totipotent cells multiply to replace those which have been lost [39].

Chemotherapy administered during organogenesis from 4 to 13 weeks of pregnancy is associated with an increased risk of miscarriage or congenital malformation. In this period tissues are highly sensitive to teratogenicity not only because they differentiate rapidly but also because damage to them becomes irreparable. The heart, neural tube and limbs are affected earlier than the palate and ear [40, 41]. Exposure to chemotherapeutics during the second and third trimester after organogenesis ends, does not usually increase the teratogenic risk.

Another factor influencing drug teratogenicity is pharmacokinetics. The increased blood volume (by almost 50%), and renal clearance during pregnancy, might for example decrease active drug concentrations [42]. If the mixed oxidase system in the liver worked faster it could also help reduce drug concentrations. And gastrointestinal functional changes may alter drug absorption [43, 44].

The third factor influencing teratogenicity is the drug class. A systematic review assessing outcomes in patients with acute myeloid leukemia during pregnancy reported 2 fetal deaths in 4 mothers receiving cytarabine and daunorubicin in the first trimester, whereas in 38 women who began therapy in the second trimester there were 3 fetal deaths and 2 malformations: adherence of the iris to the posterior cornea and hypospadias [45]. In pregnant patients under combined therapy with cytarabine and idarubicin the rate of fetal defects was 28.6% and the death rate was 12.5% [45]. In a series of seven babies whose mothers received idarubicin in the second trimester for acute myeloid leukemia one showed limb defects such as shallow sacral dimples, short digits and limbs and prominent frontal skull with macrognathia [46].

A French survey addressing the anthracylines DOX and EPI reported 20 patients with breast cancer in pregnancy treated with combined anthracycline-based chemotherapy [47]. Two pregnancies in women undergoing chemotherapy during the first trimester resulted in spontaneous abortion. One pregnancy in a woman undergoing chemotherapy in the second trimester resulted in intrauterine death and 1 newborn died 8 days after birth for no known reason. In these patients, the multiagent chemotherapy regimen included EPI [47]. Another fetus died after second trimester exposure to epirubicin, vincristine and prednisone [48].

The largest case-control observational study including 413 women from **7** European countries with a primary diagnosis of breast cancer during pregnancy later established that DOX and EPI administered during the second trimester do not increase the teratogenic risks. In a series of 197 patients, 178 received EPI (102) or DOX (76) during pregnancy and of the 386 infants born, only 9 (2%) had malformations (8 in infants exposed to chemotherapy) [49].

Other clinical studies have also shown that DOX and EPI-based chemotherapy in the second and third trimesters can be administered with minimal teratogenic risk to the developing fetus. Hence international guidelines consider these anthracycline regimens safe and should be offered according to management protocols defined by the risk of breast cancer relapse and mortality [50, 51]. Conversely, the reported idarubicin-induced teratogenicity suggests that gynecologists and oncologists should prescribe this drug only with caution when no alternative exists for pregnant mothers with cancer, especially those with hematological cancer.

#### Pregnancy complications

Second and third trimester anthracycline exposure may increase the risk of pre-term labor and low birth weight. In the French survey delivery was timed at a mean 34.7 weeks (**±** SD 2.2 weeks) with two newborns having complications related to prematurity (transient respiratory distress) [47]. In their study, Hahn et al. extended previous evidence from the MD Anderson Cancer Center [52] by reporting 57 pregnant breast cancer patients treated with fluorouracil–DOX–cyclophosphamide (FAC) in an adjuvant (n. 32) or neoadjuvant (n. 25) setting [53]. Most patients delivered at a gestational age of at least 34 weeks (average 37 weeks, range 29–42) and 28% of the newborns had difficulty in breathing [53]. A series of 103 breast cancer pregnant patients receiving anthracycline-based treatment delivered at a mean gestational age of 35.8 ± 1.9 weeks with birth weight 2836 ± 1075 g [54] whereas in 26 patients under anthracycline-based regimens, the median gestational age at delivery was 35 weeks (range 28–40 weeks) with two preterm deliveries [55].

The relationship between anthracyclines or in general chemotherapy during pregnancy and increased preterm labor is unclear: a possible contributing factor is maternal stress associated with cancer diagnosis and treatment [56]. In a retrospective study conducted in our gynecologic clinic, we suggested explaining to the mother that because many chemotherapeutic regimens including anthracyclines leave the fetus unchanged mothers should whenever possible avoid prematurity so as to reduce prematurity-induced medical complications at birth and in the long-term [57].

#### Cardiac toxicity

Cardiac toxicity is a specific feature of anthracycline toxicity [58] and emerged as a major concern after idarubicin-based chemotherapy administered during the second trimester or daunorubicin during the third trimester of pregnancy [32, 59]. The cardiac damage appears as heart dilatation, myocardial hypertrophy, dilated coronary arteries [60] or moderate right atrial and right ventricular dilatation with mildly depressed function, two small septal secundum atrial defects, and a small patent ductus arteriosus [61]. Follow-up examinations after 6 and 2 months found no residual signs of cardiomyopathy.

The safety profiles for DOX and EPI differed from those for idarubicin and daunomycin insofar as the first case reports and small case series suggested that this class of agent had no detectable effect on the fetal heart [62, 63]. Otherwise, larger retrospective studies confirmed the cardiac safety of DOX and EPI regimens, administered during the second and third trimester in adjuvant, neoadjuvant and metastatic settings [55]: even a two-week dose-dense chemotherapy induced no cardiac toxicity [64]. **S**ome studies on DOX in pregnancy reported a reduction in left ventricular wall thickness without heart defects or functional disorders [65].

Conversely, although EPI has been considered devoid of cardiotoxic effects [66–70] one report describes transient ventricular hypokinesia [71]. In another report from our gynecological institute supported by myocardial and placental evidence we showed that epirubicin cardiotoxicity had a causative role in the death of a twin who died shortly after receiving glucocorticoids for lung maturation [72]. Because glucocorticoids regulate in a complex manner the P-gp transport system at gene transcription and translation levels they could have contributed to the risk for damage from anthracycline exposure [73]. We also confirmed epirubicin cardiotoxicity by finding high troponin levels and transient left ventricular septal hypokinesia in the surviving newborn [72].

Two-dimensional echocardiography helped to evaluate fetal cardiac function including systolic and diastolic measures showing that anthracycline-based chemotherapy had no effect on ventricular diameters, septum thickness, ejection fraction and shortening fraction in the exposed fetuses [74].

In conclusion, cardiac toxicity during pregnancy or at birth therefore differs according to the type of anthracycline used. Despite rare toxicity reports [71, 72], studies in large populations [55, 64] imply that DOX and EPI have an acceptable safety profile.

#### Late cardiac toxicity

Older studies used two-dimensional echocardiography to calculate fractional shortening recommended as the best method to evaluate late cardiac toxicity in patients with cancer who received anthracyclines. This imaging technique showed that anthracyclines given for hematological malignancies during pregnancy induced no cardiac damage, even at follow-up repeated for 20 years every 5 years after birth until 29 years of age [75]. New echocardiographic equipment now under development might visualize earlier changes.

An observational study used speckle-tracking echocardiography, a recently developed technique for characterizing and quantifying myocardial deformation, in children >5 years old whose mothers had received anthracyclines during pregnancy. In this case-control study this technique found the use of anthracyclines for hematological malignancies during pregnancy was not associated with differences between case and control groups in fraction shortening, interventricular septum, left ventricular posterior wall, left ventricular end-diastolic diameter, left ventricular ejection fraction, and right ventricular end-diastolic diameter [76].

In an important multicenter observational case-control study assessing children who were prenatally exposed mainly to anthracycline-based chemotherapy at birth (53 children), at age18 months, 5–6, 8–9, 11–12, 14–15, or 18 years, ECG measurements detected neither arrhythmia nor conduction abnormalities. The echocardiographic examination found a clinically smaller but statistically significant ejection fraction, fraction shortening and interseptal thickness decrease in the patient group than in the control group [77]. All patients were within the normal ECG range without any structural defects. Similarly, in another study, the echocardiographic assessment at 36 months in 47 children exposed to anthracyclines, failed to show signs of early cardiac remodeling, with normal wall thicknesses and chamber dimensions, and all systolic and diastolic function measurements came within normal ranges [78].

#### Late neurodevelopmental toxicity

As a gatekeeper for protecting the central nervous system, the cerebral microvessel endothelium forms the blood-brain barrier (BBB). Data acquired in recent years suggest that the BBB is active since fetal life and efficiently protects the immature brain [79]. In addition to the cellular level, several molecular mechanisms help to enhance selectivity in permitting materials to pass from the blood into the CNS. P-gp at the BBB can exert a profound effect on the ability of some drugs such as cardiovascular drugs to enter the brain. Abnormal activity involving the signaling pathways responsible for inducing functional efflux protection in blood-brain interfaces could induce influential changes in how the fetal brain is exposed to undesired substrates, eventually preventing the brain from developing normally. [80].

The existence of a protective barrier explains reassuring data suggesting that anthracyclines administered during pregnancy leave fetal neurodevelopment unchanged. After a median 18.**7-**year follow-up, anthracyclines administered for hematological malignancies during pregnancy had no effect on growth, development, hematological, cytogenetic, neurological, psychological variables, and learning disorders [81]. The aforementioned multicenter observational case-control study starting from 18 months, used clinical neurological examinations and tests assessing the general cognitive functioning level (Bayley or intelligence quotient [IQ] test) on a child cohort. At 5 years tests were completed by audiometry, the Auditory Verbal Learning Test, and Children’s Memory Scale subtasks, and the Test of Everyday Attention for Children. At a median 24-month follow-up average IQ score**s** increased by 11.1 points for each month increase in pregnancy duration but neurocognitive outcomes were within normal ranges [77]. In a small group**,** most children exposed prenatally to anthracycline-based chemotherapy exhibited no significant differences in cognitive ability, school performance, or behavioral competence [82].

In conclusion, although late anthracyline-induced cardiac and neurological toxicity raise little concern, patients need long-term follow-up to detect subtle changes during life before they become more severe.

## Conclusion

Cancer in pregnancy is a clinical and ethical dilemma. The care provider has to manage two lives: the life of a woman suffering from a disease that if left untreated is likely to progress, and the life of a fetus exposed to damage from maternal chemotherapies administered.

Pregnant women are reluctant to receive chemotherapy because they fear its possible deleterious effects on the developing fetus even though current research implies that the placenta acts as a barrier thereby guaranteeing low transplacental anthracycline transfer.

Convincing evidence shows that chemotherapy given during the second and third trimesters of pregnancy is safe but anthracycline-based chemotherapy regimens raise concerns related to fetal cardiac toxicity.

Anthracyclines induce cardiotoxicity in the fetus first because fetal cardiac tissue is especially vulnerable to free radical damage owing to low cardiomyocyte tolerance to changes in the oxidant-antioxidant system, and also because embryonic and fetal myocytes grow and develop fully only later during prenatal life. Hence, fetuses exposed to anthracycline during pregnancy might later become patients at risk for premature cardiovascular disease during adulthood.

Despite growing evidence that anthracycline-based therapy is safe and effective, certain anthracycline drug classes continue to raise doubts. Whereas DOX and EPI have acceptable safety profiles, reported idarubicin teratogenicity warns against its use for pregnant mothers with cancer, especially hematologic cancer, unless no other option exists.

Hence pregnant women receiving anthracycline-based chemotherapy should undergo regular diagnostic imaging with the newly available techniques to detect damage early, and allow alternative therapeutic options. While we await new research to dispel uncertainty on the potential long-term effects of in utero anthracycline, patients need regular follow-up to detect late, subtle anthracyline-induced cardiac and neurological toxicity changes over time.

An equally important concern for obstetricians and gynecologists is that when pregnant women receive drugs that inhibit or induce P-gp, drug interactions may alter placental P-gp barrier function and subsequently change fetal exposure. Hence, giving glucocorticoids to prevent lung immaturity may hamper placental EPI metabolism, ultimately increasing its toxicity by reducing Pgp expression on the placental surface.

Some fetuses, such as those with intrauterine growth retardation, are more vulnerable and require more intensive monitoring owing to factors associated with a compromised placental supply of nutrients and oxygen that involves altered metabolic changes in pregnancy, and chronic inflammation. These babies may have an increased risk for damage from anthracycline exposure.

Anthracycline exposure may increase the risk of pre-term labor. To minimize prematurity-induced complications, doctors who care for pregnant women should avoid inducing labor without any obstetrical indications.

Knowing more about the biological pathways regulating placental transport during fetal development and understanding how oxidative stress, inflammation and drugs modulate them, may open the way for tailored drug administration. For example, in animal experiments, when low P-gp-induced expression caused severe oxidative injuries in placentas from the fetal cardiac rat model, giving vitamin C reduced oxidative stress and rebuilt placental protective mechanisms [83].

P-gp at the BBB can prevent some drugs from entering the brain. Studying the BBB may open up new opportunities for tailoring anticancer therapy to improve or restore neuroprotective functions in the blood-brain interfaces thus preventing intrauterine injuries from anticancer therapy.

Finally, analyzing the pharmacokinetic features underlying gestation-induced changes will make it easier to adjust chemotherapy dose regimens.

## Abbreviations

ABVD: DOX-bleomycin-vinblastine-dacarbazine
BBB: Blood-brain-barrier
BCRP: Breast cancer resistance protein
DOX: Doxorubicin
EPI: Epirubicin
FAC: Fluorouracil-DOX-cyclophosphamide
FEC: Fluorouracil-EPI-cyclophosphamide
NADPH: Nicotinamide adenine dinucleotide phosphate
PARP: Poly(ADP-ribose) polymerase
P-gp: P-glycoprotein
PL: Pegylated
